# A new method for measuring wetness of flowing steam based on surface plasmon resonance

**DOI:** 10.1186/1556-276X-9-18

**Published:** 2014-01-13

**Authors:** Xinjiang Li, Xiaofeng Li, Chinhua Wang

**Affiliations:** 1 Institute of Modern Optical Technologies & Collaborative Innovation Center of Suzhou Nano Science and Technology, Jiangsu Key Lab of Advanced Optical Manufacturing Technologies & MOE Key Lab of Modern Optical Technologies, Soochow University, Suzhou 215006, China; 2 School of Physical Science and Technology, Soochow University, Suzhou 215006, China

**Keywords:** Stream wetness, Surface plasmon resonance, Kretschmann configuration

## Abstract

A novel method for real-time and inline wetness measurement based on surface plasmon resonance (SPR) is presented in this paper. The Kretschmann geometry is adopted to excite surface plasmon waves in our measurement system. In order to prevent water coating, an ultrathin layer of hydrophobic coating is formed on the surface of Au layer. The experimental results show that the level of steam wetness can be obtained via the area ratio of water and air on the prism, which is determined by analyzing the SPR spectrum of wet steam based on a Gaussian model. In addition, during the online measurement of flowing wet steam wetness, significant shifts in the resonant position of the SPR spectrum occurred, which can be attributed to the strong interaction of the adjacent water droplets.

## Background

Interest in wet steam research was sparked by the need for efficient steam turbines used in power generation. The subject has become increasingly important in the current decade with the steep increase in fuel cost. Since the 1970s, wetness measurement technology has made a great progress. Although with a simple principle, thermodynamic method has its disadvantages, such as a long measuring period and large error [1,2]. Optical method, primarily based on light scattering techniques and microwave resonant cavities, has a high measuring precision, however, with the estimation of steam quality strongly depending on the droplet size classification [3-5]. Electrostatic charge and capacitance methods are new with rare studies on electrostatic charge of droplets in wet steam flow in low-pressure steam turbines [6-8]. For its high precision, tracer determination method is popular in nuclear power plants, but there are several adverse aspects such as complicated operating process, intricate data processing, and costly instruments [9,10]. Therefore, up to now, online measurement of wetness in steam turbines as accessibility is still a major challenge.

## Methods

In this paper, we consider the use of surface plasmon resonance (SPR) for measuring steam wetness. Surface plasmon (SP) waves have been studied since the 1960s. They can be described as a collective oscillation in electron density at the interface of metal and dielectric. Resonance occurs when the wave vector of surface plasmon wave equals to the tangential component of evanescent wave vector (i.e., the phase-matching condition) under appropriate incident conditions (e.g., incident angle and wavelength). Under SPR, the incident light will be strongly absorbed, showing a deep reflection dip. Since a stringent phase-matching condition is needed, SPR is very sensitive to the system configuration and surrounding environment, which allows using this unique property for measuring steam wetness. According to the dielectric theory, at room temperature, the relative dielectric constant of saturated water vapor is close to that of air. Therefore, the wet steam is modeled by spraying atomized water on the hydrophobic coating layer of the Kretschmann configuration with the designed two-phase nozzle in the experiments. The steam wetness is regulated through the spraying quantity, and the absolute wetness *X* is given by [1,2,8]

(1) $$X=\frac{\rho_{w}\emptyset_{w}}{\rho_{w}\emptyset_{w}+\rho_{g}\emptyset_{g}}\times 100\%$$

where ∅ _w_(*ρ*_w_) and ∅ _g_(*ρ*_g_) are the volume ratios (densities) of spraying water and air flow, respectively. Assuming a constant transverse (parallel to the metal-dielectric interface) droplet distribution, Equation 1 can be simplified as

(2) $$X=\frac{\rho_{w}S_{w}}{\rho_{w}S_{w}+\rho_{g}S_{g}}\times 100\%$$

where *S*_w_ and *S*_g_ are the area ratio of water and air on SPR surface, respectively. Here, *S*_w_ and *S*_g_ can be measured by SPR.

**Figure 1:**
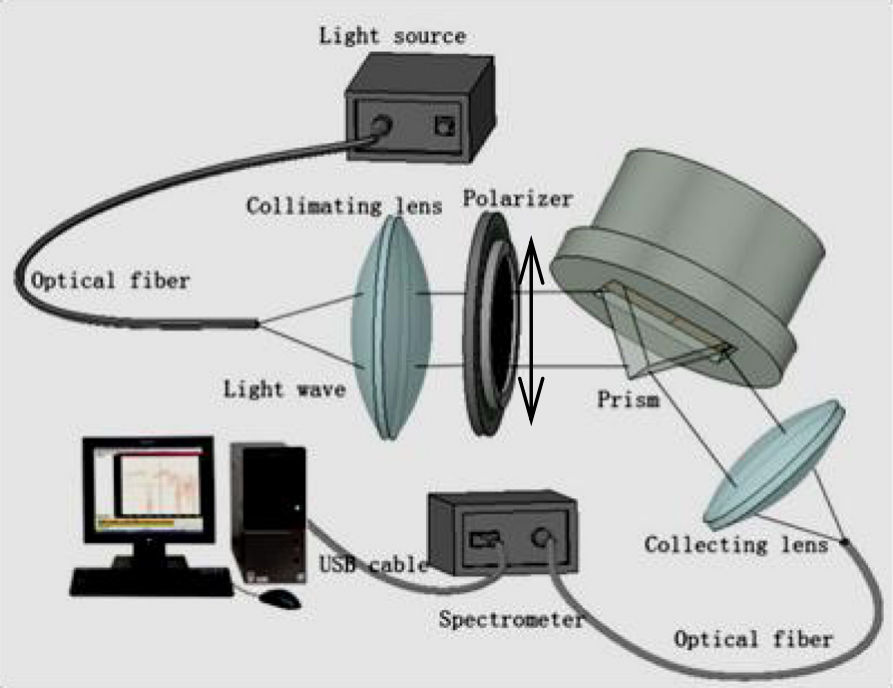
Experimental arrangement.

A schematic of the steam wetness measurement system is displayed in Figure 1, which is composed mainly of transmitter, measuring space, and receiver:

1. The transmitter unit is configured to convert the light source into a parallel light beam with transverse magnetic polarization (i.e., the magnetic field direction parallel to the metal/prism surface in Kretschmann configuration). It comprises the DH-2000 Deuterium Tungsten Halogen Light Source (Ocean Optics, Dunedin, FL, USA), optical fiber, lens, and polarizer.

2. The core component of measuring space is the Kretschmann configuration, also referred to as attenuated total reflection, in which a 45-nm Au layer is evaporated on top of a SF2 prism. In order to prevent water coating, a 2- to 3-nm ultrathin layer of hydrophobic thiol coating is formed on the surface of the Au layer. In our experiments, the special container on top of the Kretschmann configuration is designed to hold water.

3. The receiver, which is composed of lens, optical fiber, and spectrometer, accepts the reflected light and couples it to our spectrometers.

The sensing application of the SPR system can be realized by modulating either the wavelength or incident angle [11]. The controlling of light injection angle requires a fine adjustment of the physical configuration precisely; therefore, we choose to implement such a wetness sensing through controlling and analyzing the reflection spectrum under SPR, i.e., wavelength modulation surface plasmon resonance. Since under different incident angles, SPRs occur in different wavelengths, we fix the incident angle to be 69.3° which simplifies the system as well as provides high enough sensitivity.

## Results and discussion

**Figure 2:**
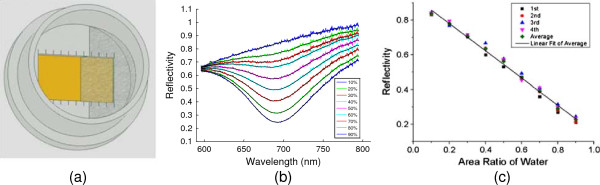
**Schematic and results of the measurement system with top surface partially immersed in water. (a)** Schematic of top view of the measurement system. **(b)** SPR spectra under various immersion percentages. **(c)** Dependence of the reflectivity at 693 nm against the immersed area: (dots) experimental data and (line) linear fit.

We first focus on the case where part of the top surface area of a rectangular prism is immersed in water (see Figure 2a). The reflection spectra under different immersion percentages are measured and plotted in Figure 2b, which actually exhibits the spectral response of SPRs contributed from both water-Au and air-Au interfaces. However, according to our calculation, under an identical injection angle, SPR excited from air-Au interface occurs at a much shorter wavelength that is beyond the scope of our spectrometer; thus, the dips observed in Figure 2b are mainly from the Au-water interface. From this measurement, the adjustment of immersion ratio leads to a substantial change of the reflectivity (especially at the SPR dip at around 693 nm), however, without shifting the resonant wavelength noticeably. This further confirms that the SPR is primarily from a given metal-dielectric interface (i.e., water-Au interface); the variation of the surface areal coverage modifies the portion of incident light to couple into the SPR, therefore resulting in a significant change of the dip reflectivity. From the varying dip reflectivity, the coverage of water or air can be estimated. The corresponding calibration curve for the reflectivity of SPR peak is shown in Figure 2c. The SPR reflectivity follows a linear decrease with the gradually increased immersion area. A linear fitting indicates that the adjusted *R* squared is about 0.9959. The error term comes mainly from uncertainty of our immersed area calibration and measurement noise and can be further reduced with an optimized experiment setup.

**Figure 3:**
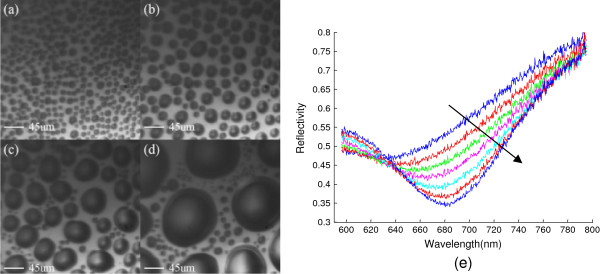
**Distributions of water droplets and corresponding SPR spectra. (a, b, c, d)** Distributions of water droplets on the SPR system with continuously spraying wet steam onto the sensor surface. **(e)** The corresponding SPR spectra.

Figure 3a,b,c,d illustrates the measured surface patterns, where the size and distribution information of water droplets can be achieved, with wet steam continuously spraying on the hydrophobic coating layer. Due to hydrophobic coating, the wet steam does not form a continuous water coating on the sample surface; instead, a number of isolated water droplets exist. The real-time SPR spectrum of wet steam is recorded online with continuous spraying (Figure 3e). Unlike the SPR spectra shown in Figure 2b where the prism is immersed in water, distinct changes in both resonant position and reflected light intensity are observed. With large droplets formed, the resonant peak shifts to a longer wavelength and finally reaches the SPR wavelength of water-Au system. The changes in intensity can be understood to be from the size variation of water droplets. Intuitively, the intensity is related to the surface area covered by water droplets. Meanwhile, the shift of the resonance can be attributed to the interaction of the droplets on top of the surface.

According to the dispersion relation of SPR, the effective permittivity of air droplet (two phases) composition can be obtained without a doubt. There exist several theories which can calculate the effective permittivity of such mixtures. One of the most widely used formulations is the Maxwell Garnett (MG) theory [12]. Unfortunately, MG theory and other dielectric mixture theories [13] are useful only for the case when the gap size between the droplets is far less than the effective wavelength. Notice here that the ratio of gap size of the adjacent droplets to effective wavelength of SP is between 10^1^ and 10^2^; therefore, the steam wetness cannot be simply derived from the summation of the two-phase behavior.

**Figure 4:**
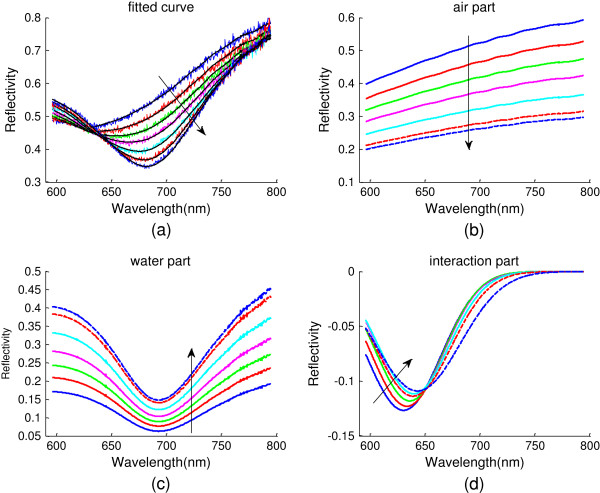
**Measurement and decomposition curves.** Measured SPR curves **(a)** and their decompositions: air **(b)**, water **(c)**, and interaction **(d)** contributions.

In our experiment, the SPR spectrum of wet steam is actually a contribution of three parts: air, droplets, and their mutual interaction. By analyzing the curves in Figure 3e, we find that all curves have a Gaussian line shape, which allows us to use a Gaussian model to post-process the experimental results. As measured above, the line shape of the SPR spectrum for air-Au or water-Au system does not change for a fixed incident angle. Thus, the SPR curve of wet steam can be reasonably decomposed into air, water, and interaction parts. Applying a similar technique for all curves in Figure 3e, we can well fit the experimental measurements analytically as shown in Figure 4a. Figure 4b,c shows the fitted curves for air-Au and water-Au contributions, respectively. It should be noted that the reflectivity of the air part decreases while that of the water part increases along the arrow direction. This seems to conflict with the finding of Figure 2b, where increased water ratio leads to reduced reflectivity. However, we would like to emphasize that the spectral response shown in Figure 2b is a whole effect contributed from both water-Au and air-Au portions as discussed previously. On the contrary, those shown in Figure 4b,c exhibit the respective contribution. In fact, Figure 4b has displayed a substantial reflection decrease which can overcome the slight increase contributed from water-Au. A further explanation of such a slight reflectivity increase shown in Figure 4c is as follows: with more area covered by water, the reflection contribution from the water-Au portion is increased in a straightforward way (despite the dips excited from SPR). In addition, the intensity of interaction part also decreases when water coverage is increased, and a significant redshift of the peak position is observed, as shown in Figure 4d, which well explains the resonance shift observed in Figure 3. The negative contribution from the interaction reflects that the mutual interaction of the adjacent droplets causes additional loss to the reflected light. As the gap between the adjacent droplets becomes larger with an increase in droplet size, the interaction is weakened, leading to a weaker contribution from the interaction to the total spectral response (see Figure 4d).

**Figure 5:**
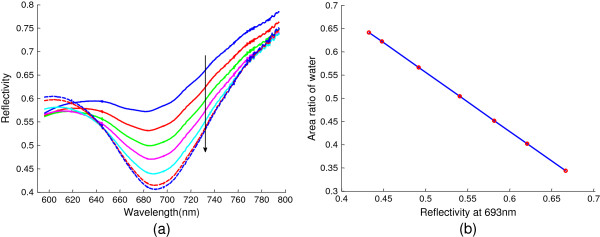
**Superposition curves and measured area ratios. (a)** Superposition curves of Figure 4b,c and **(b)** measured area ratios of water on the sensor surface.

Compared to Figure 2b, the reflectivity shown in Figure 4b,c just directly reflects the respective area ratio of air and water droplets on the prism (in other words, they are isolated without interaction),while the curves in Figure 2b include the contributions from both air and water portions, which means that the reflectivity in Figure 2 is the sum of air and water parts under appropriate weighting factors. Therefore, superposition has to be adopted in order to estimate the wetness in an accurate way. Figure 5a shows the superposition curves of Figure 4b,c. Consequently, according to the calibration curve above, we can get the area ratio of water droplets in different wet steam statuses (see Figure 5b which enables the calculation of the absolute wetness through Equation 2).

## Conclusions

We demonstrate a novel method for wetness measurement based on surface plasmon resonance. The obtained SPR spectrum of wet steam is analyzed by a Gaussian model. From this analysis, the area ratio of water and air via the reflectivity of SPR spectrum of wet steam is determined, and the wetness of wet steam can be obtained. Moreover, a clear shift in the resonant position of SPR with continuously spraying wet steam is observed and has been tentatively ascribed to interaction between adjacent droplets.

## Abbreviations

MG: Maxwell Garnett; SP: Surface plasmon; SPR: Surface plasmon resonance.

## Competing interests

The authors declare that they have no competing interests.

## Authors' contributions

XJL carried out the experiments and drafted the manuscript. Discussion and revision were from XFL and CHW. XFL improved the manuscript. CHW supervised the work. All authors read and approved the final manuscript.
